# Case report: Traumatic hemorrhagic cervical myelopathy in a dog

**DOI:** 10.3389/fvets.2023.1260719

**Published:** 2023-10-05

**Authors:** Koen M. Santifort, Ines Carrera, Simon Platt

**Affiliations:** ^1^IVC Evidensia Small Animal Referral Hospital Arnhem, Neurology, Arnhem, Netherlands; ^2^IVC Evidensia Small Animal Referral Hospital Hart van Brabant, Neurology, Waalwijk, Netherlands; ^3^Vet Oracle Teleradiology, Norfolk, United Kingdom

**Keywords:** hemorrhage, hematomyelia, spinal cord, recovery, durotomy, short-term outcome

## Abstract

A 1.5-year-old female entire French bulldog was referred for neurological evaluation, further diagnostic tests, and treatment 24 h after a road traffic accident. Initial emergency treatment, diagnostic tests, and stabilization had been performed by the referring veterinarian. Neurological examination revealed severe spastic non-ambulatory tetraparesis and was consistent with a C1-5 myelopathy. A magnetic resonance imaging (MRI) study revealed an irregular to elongated ovoid intramedullary lesion centered over the body of C2. The lesion showed marked signal heterogeneity with a central T2W and T2* hyperintense region, surrounded by a hypointense rim on both sequences. The lesion appeared heterogeneously T1W hypointense. The lesion was asymmetric (right-sided), affecting both white and gray matter. The C2-3 intervertebral disk appeared moderately degenerate with a Pfirrmann grade of 3. No evidence of vertebral fracture or luxation was found on radiographs or MRI of the vertebral column. Additional soft tissue abnormalities in the area of the right brachial plexus were suggestive of brachial plexus and muscle injury. A diagnosis of traumatic hemorrhagic myelopathy at the level of C2 and concurrent brachial plexus injury was formed. Conservative treatment was elected and consisted of physiotherapy, bladder care with an indwelling urinary catheter, repeated IV methadone based on pain scoring (0.2 mg/kg), oral meloxicam 0.1 mg/kg q24h, and oral gabapentin 10 mg/kg q8h. The dog was discharged after 4 days, with an indwelling urinary catheter and oral medication as described. The catheter was replaced two times by the referring veterinarian and finally removed after 10 days. Thereafter, voluntary urination was seen. During the 2 months after the road traffic accident, slow recovery of motor function was seen. The right thoracic limb recovery progressed more slowly than the left limb, also showing some lower motor neuron signs during follow-up. This was judged to be consistent with a right-sided brachial plexus injury. The dog was reported ambulatory with mild residual ataxia and residual monoparesis of the right thoracic limb at the last follow-up 3 months post-injury. This case report highlights the MRI-based diagnosis of traumatic hemorrhagic myelopathy in a dog. A fair short-term outcome was achieved with conservative treatment in this case.

## Introduction

Hemorrhagic myelopathy, also called hematomyelia, in dogs can be due to various etiologies. These include trauma associated with vertebral fractures/luxation [e.g., due to road traffic accidents (RTA)], trauma associated with congenital anomalies of the craniocervical region (e.g., odontoid process malformation and atlantoaxial instability), iatrogenic trauma (e.g., due to spinal cord puncture during cerebrospinal fluid taps), vascular malformations (e.g., arteriovenous malformation), intervertebral disk disease (e.g., intervertebral disk extrusion), neoplasia (e.g., metastatic hemangiosarcoma or lymphoma), inflammatory disease (e.g., steroid-responsive meningitis arteritis), and hemorrhagic diathesis (e.g., related to *Angiostrongylus vasorum* infections) (1–9). When no causes are identified, the terms primary hematomyelia or idiopathic hemorrhagic myelopathy may be applicable (9, 10).

In human medical literature, hemorrhages can be found in the spinal cord in cases of spinal cord injury (SCI) without radiographic abnormalities (SCIWORA) (11–15). This is defined as SCI without evidence of vertebral fractures or dislocation based on radiographic studies. Since this is a fairly rare clinical entity, much of its exact pathophysiology remains unknown. Clinically, human patients (often children) are presented with various degrees of neurological dysfunction (12–14). In one systematic review, “complete” SCI defined as a lack of motor and sensory function “below” the level of the lesion in the spinal cord was reported at initial presentation in almost 20% of patients (12). Recent studies have provided evidence for improved outcomes following early surgical intervention (15). No such studies are available regarding clinical canine patients.

In this case report, we describe the magnetic resonance imaging (MRI) based diagnosis of traumatic hemorrhagic cervical myelopathy in a dog without evidence of vertebral fractures or luxation.

## Case description

**Figure 1:**
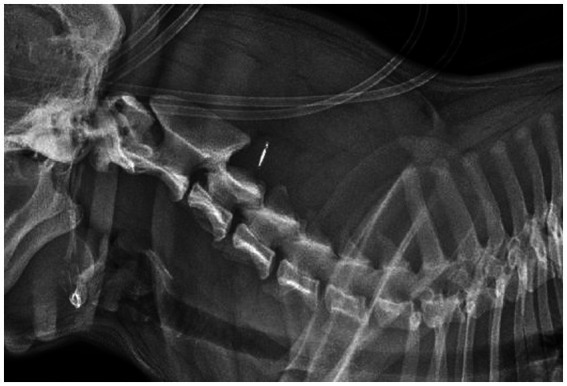
Radiograph (laterolateral, right-sided recumbency) of the cervical vertebral column.

A 1.5-year-old female entire French bulldog was referred for neurological evaluation, further diagnostic tests, and treatment 24 h after a road traffic accident (RTA). The dog was chasing a cat and collided with a moving vehicle head-on, hitting the side of that vehicle. Bystanders reported that the dog immediately collapsed. The dog was rushed to the nearest veterinary practice. The dog was presented there in lateral recumbency with increased extensor tone of all four limbs. Mucous membranes were noted to be slightly blueish. No voluntary movement of limbs was recorded at that time, consistent with spastic tetraplegia. The dog was noted to show reduced responsiveness, and a modified Glasgow coma scale (MGCS) score of 9 was recorded without further details. Initial emergency treatment, diagnostic tests, and stabilization were performed. This included an IV bolus of 15 mL/kg 0.9% sodium chloride, followed by 20 mL/kg/h Ringers solution, and a single IV bolus of 1 g/kg mannitol [based on concerns for increased intracranial pressure (ICP)]. For analgesia, several boluses of methadone (0.2 mg/kg IV) had been administered. Heart rate increased to 70–90 beats/min over the next hour and the dog became more responsive, with a MGCS score of 13, increasing to 18 during the rest of the day on repeat examinations. Pulse oximetry consistently showed a SpO_2_ of 99–100%. Further diagnostic tests included hematology (no significant abnormalities), biochemistry [hyperglycemia (11.72 mmol/L, reference range 4.11–7.95)] and increased blood lactate (5.26 mmol/L, reference range 0.50–2.50), ultrasound of the thorax and abdomen (possible signs of right-sided lung contusion), laterolateral radiographs of the thorax (signs suggestive of lung contusion), cervical vertebral column (Figure 1), and thoracolumbar vertebral column, and non-invasive blood pressure measurements (80–115 mmHg). Repeat testing of blood lactate showed values within the reference range. When the patient was stable the next day, the owners opted for a referral for further neurological examination, diagnostic testing, and treatment.

During the 2-h drive to the referral hospital, the dog had become hyperthermic (rectal temperature of 40.5 degrees Celsius) and was stabilized by the emergency department. Treatment at that point included active cooling, oxygen supplementation via nasal catheter and flow-by. After achieving normothermia, neurological examination revealed severe spastic non-ambulatory tetraparesis, worse in the thoracic limbs than the pelvic limbs. There was some voluntary movement of the limbs, more so in the pelvic limbs than the thoracic limbs. Spinal reflexes were intact in the pelvic limbs but decreased in the thoracic limbs on both sides. These findings were deemed consistent with a C1-5 myelopathy (likely involving the central cord). After discussion with the owners, an MRI study of the cervical spinal cord was performed, including bilateral brachial plexus regions. Additional imaging studies including an MRI of the brain and computed tomography (CT) of the vertebral column, thorax, and abdomen were declined due to financial restrictions. MRI sequences included T2-weighted (T2W) fast-spin echo (FSE) sagittal plane, T1W FSE sagittal plane, short-tau inversion recovery (STIR) sagittal plane, STIR dorsal plane, 3D fast gradient echo combined with water excitation technique (FFE3D combined with WET), T2W FSE transverse plane, T1W FSE transverse plane, T2*W gradient echo transverse plane, and 3D T1W magnetization prepared—rapid gradient echo (MPRAGE) sagittal plane post-contrast.

**Figure 2:**
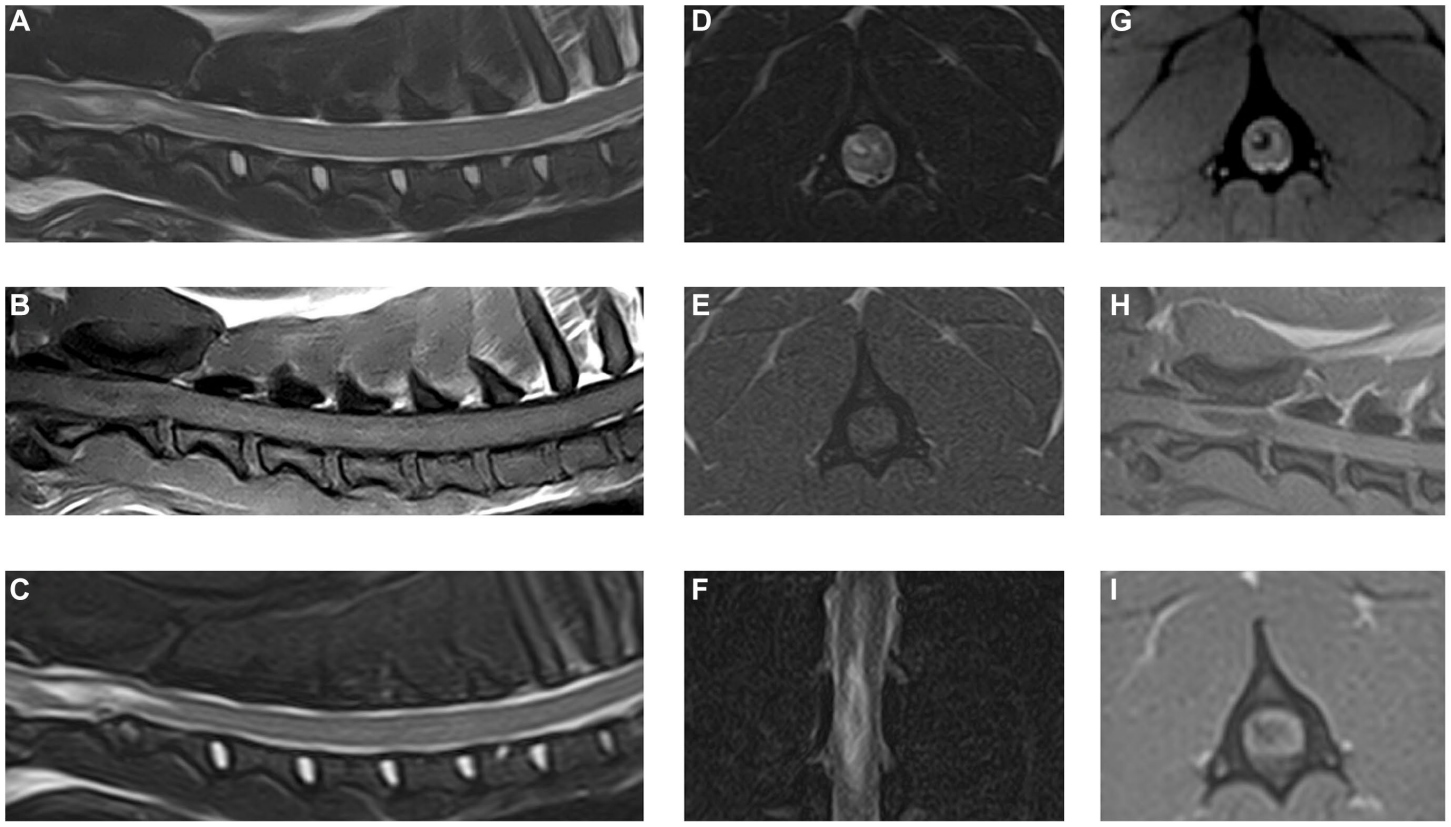
Magnetic resonance images of the cervical spinal cord and surrounding structures. **(A)** T2W sagittal plane, **(B)** T1W sagittal plane, **(C)** STIR sagittal plane, **(D)** T2W transverse plane at the level of C2 vertebral body, **(E)** T1W transverse plane at the level of C2 vertebral body, **(F)** STIR dorsal plane, **(G)** T2*W transverse plane at the level of C2 vertebral body, **(H)** 3D T1W MPRAGE sagittal plane, and **(I)** 3D T1W MPRAGE transverse reconstruction at the level of C2 vertebral body.

The MRI study revealed an irregular to elongated ovoid intramedullary lesion centered over the body of C2 (Figure 2). The lesion showed marked signal heterogeneity with a central T2W and T2* hyperintense region, surrounded by a hypointense rim on both sequences. The lesion appeared heterogeneously T1W hypointense. The lesion was predominantly right-sided and dorsolateral within the spinal cord, affecting both white and gray matter. The C2-3 intervertebral disk appeared moderately degenerate with a Pfirrmann grade of 3. No evidence of vertebral fracture or luxation was found on radiographs of the vertebral column or the MRI study. Additional findings in the area of the right brachial plexus were suggestive of brachial plexus and muscular injury. A diagnosis of traumatic hemorrhagic myelopathy at the level of C2 and concurrent brachial plexus injury was formed.

Coagulation tests (activated partial-thromboplastin time and prothrombin time) were within the reference range. Repeated clinical examinations, focusing on signs or evidence of hemorrhagic diatheses (e.g., hemorrhages in mucous membranes) did not reveal any abnormalities. Conservative treatment was elected and consisted of fluid therapy, physiotherapy, an indwelling urinary catheter, repeated non-invasive blood pressure measurements, repeated IV methadone boluses based on pain scoring (0.2 mg/kg), oral meloxicam 0.1 mg/kg q24h, and oral gabapentin 10 mg/kg q8h.

Over the next 4 days, progressive signs of recovering motor function were observed in the left thoracic limb, both pelvic limbs, and, to a lesser degree, the right thoracic limb. Withdrawal reflexes were normal in the left thoracic and both pelvic limbs at that point but decreased in the right thoracic limb. Extensor muscle tone in all limbs had decreased, most notably in the right thoracic limb. The dog was discharged after 4 days, with an indwelling urinary catheter and oral medication as described. The catheter was replaced twice by the referring veterinarian and finally removed after 10 days. Thereafter, voluntary urination was seen. Meloxicam was discontinued a week later, and gabapentin was tapered and discontinued 2 weeks later. During the 2 months after the road traffic accident, slow recovery of motor function was seen. The right thoracic limb recovery progressed more slowly than the left limb, also showing some lower motor neuron signs (e.g., flaccid paresis) during follow-up at the referring veterinarian and was also visible on videos sent by the owners for remote evaluation by the neurology department of the referral hospital. The recovery was judged to be consistent with right-sided lateralization of the spinal cord hemorrhage and involvement of the brachial plexus on the right. The dog was reported to be ambulatory with mild residual ataxia and moderate to severe monoparesis of the right thoracic limb at the last follow-up 3 months post-injury. Physiotherapy including hydrotherapy had been commenced by the owners and is being continued long-term with the aid of orthopedic braces for the right thoracic limb. The owners were happy with the outcome at the time of writing and the patient remains under the care and supervision of the referring veterinarian.

## Discussion

This case report describes the neurological presentation, MRI findings, conservative management, and short-term outcome of traumatic hemorrhagic cervical myelopathy in a French bulldog. Although the presenting clinical signs were severe directly after the traumatic event (RTA in this case), emergency treatment followed by conservative treatment for the cervical myelopathy and suspected brachial plexus injury resulted in a fair short-term outcome.

For the treatment of traumatic SCI in dogs, it is of vital importance to account for basic support measures and general stabilization before focusing on neurological signs and prognostication (15). In the case reported here, initial stabilization was performed by the referring veterinarian. The dog was transported for further work-up when it was deemed to be stabilized. However, hyperthermia developed during the drive to the referral hospital, and measures needed to be taken to stabilize the patient. Fortunately, there did not seem to have been any neurological deterioration as determined by the results of the neurological examination vs. the descriptions of the referring veterinarian.

As emphasized in several veterinary texts, maintaining adequate tissue (spinal cord) perfusion is a key factor in the treatment of traumatic SCI in dogs (16–18). Spinal cord perfusion pressure (SCPP) is not routinely clinically measured [i.e., calculated from measurements of intradural or “intraspinal” pressure (ISP) and mean arterial blood pressure (MAP)] in canine traumatic SCI cases. Human literature, reporting results in clinical patients, has shown that such measurements can contribute to guiding more efficient and objective treatment measures (19, 20). Even if SCPPs are not performed, maintaining blood pressure (MAP) within the reference range can be and is regarded as a cornerstone of treatment (16, 17). This makes good sense, as low blood pressure will be detrimental to SCPP. High blood pressure is also to be avoided, as the blood-spinal cord barrier (BSCB) and local vascular autoregulation in the injured spinal cord is affected (16–23).

Numerous other possible avenues of treatment are discussed in veterinary as well as human literature (16–18, 23). These include, but are not limited to stem cell therapy, hypothermia, pharmacological treatment (e.g., the dubious role of corticosteroids), and surgery. The role of the latter deserves specific attention based on recent veterinary literature and human literature. As an option for maintaining or increasing SCPP, surgery would provide a way of removing restrictions to the expansion of the spinal cord parenchyma and/or removing compressive lesions. That is to say, surgical opening of the vertebral column and durotomy can provide the spinal cord with space to expand in case of swelling, causing a direct decrease in ISP. Indeed, the role of durotomy in canine spinal cord injury due to intervertebral disk extrusion (IVDE) is the subject of recent studies (24–27). These studies have reported positive effects on outcomes in dogs with severe grades of thoracolumbar spinal cord dysfunction. Durotomy and duraplasty has been studied in humans with SCIWORA as well (15, 20, 28). Myelotomy has also been described in animal models of SCI and is sporadically reported with positive effects in humans (28). Other types of surgery, including stabilization procedures, are reported for the management of SCIWORA in humans as well (29).

With regard to pharmacological treatment, the dog reported here received a variety of intravenous medications and fluids as emergency treatment which included mannitol and methadone. At that point in time, the diagnosis of traumatic hemorrhagic cervical myelopathy had not yet been reached. Importantly, and justly, treatment was focused on stabilizing the trauma patient (16). After the diagnosis of traumatic hemorrhagic cervical myelopathy and concurrent traumatic brachial plexus injury was determined, treatment consisted of fluid therapy, physiotherapy, an indwelling urinary catheter, repeated non-invasive blood pressure measurements, repeated IV methadone boluses based on pain scoring, oral meloxicam, and oral gabapentin. The latter three medications were used with the aim of providing adequate analgesia. Non-steroidal anti-inflammatory drugs (NSAIDs), or cyclooxygenase (COX) inhibitors are particularly preferred for the treatment of SCIWORA in human patients based on animal models and clinical experience (23). However, there are no large prospective, blinded studies assessing the effect of NSAIDs or COX inhibitors in clinical human patients, let alone canine patients with SCIWORA. Nevertheless, the use of these medications would be supported by the importance of inflammatory cascades in SCI pathophysiology as well as the need for analgesia in trauma patients in general (8, 16, 17, 23). Monitoring for side effects, such as gastrointestinal complications, is always advisable. Specifically when corticosteroids have been administered preceding or concurrently. The use of mannitol for SCI is debatable, but as SCPP would benefit from increased blood volume as well as reduction of spinal cord edema, a beneficial effect is not excluded. Future studies may provide the opportunity to develop more evidence-based guidelines. In this trauma patient, mannitol was administered due to concerns of increased ICP. Finally, the use of opioid receptor agonists may be detrimental for SCI patients. Indeed, the use of opioid receptor antagonists (such as naloxone) has shown some positive results in SCI models (16, 23). However, the use of opioid antagonists is not recommended as it would prevent adequate analgesia for a trauma patient such as reported here, and its effectiveness is not adequately supported by evidence in clinical (human or canine) acute trauma patients.

While hemorrhage is a common feature of traumatic SCI in humans in general (30), it is only reported in a minority of cases of cervical SCIWORA in humans (31–33). Up to 67% of human cases of traumatic SCI have signs compatible with spinal cord hemorrhage on MRI (30). In contrast, one study including 59 patients with SCIWORA described MRI findings consistent with hemorrhage in only two cases (31). The finding of an intramedullary hemorrhagic component is considered a robust indicator of irreversible injury and predictor of injury severity (32). Indeed, intramedullary changes including hemorrhage have been shown to negatively affect prognosis in human SCIWORA patients (12). That being said, conservative management of cervical SCIWORA with hematomyelia may still lead to a positive outcome (33). In our case, conservative management resulted in a fair outcome in the short-term, where most of the remaining deficits affected the right thoracic limb. Those deficits were best explained by a concurrent brachial plexus injury, rather than the traumatic cervical hemorrhagic myelopathy in this dog.

In dogs, the use of T2* gradient echo sequences has been reported of value in assessing hemorrhagic lesions of or affecting the spinal cord (34). In the case reported here, this sequence was instrumental for the identification of an intraparenchymal hemorrhage. No signs of linear tracts extending into the spinal cord were found and the lesion epicenter was located over the C2 vertebral body, making an intradural/intramedullary disk extrusion unlikely. There were no signs of vertebral fractures or dislocation on laterolateral radiographs or the MRI study. However, it has been reported that MRI is less sensitive than CT in the identification of vertebral fractures (35). Orthogonal radiographs also have limited sensitivity (36), let alone single-view radiographs which only provide a two-dimensional evaluation of the vertebral column as was available in this case. Thus, a limitation to this case report is the lack of orthogonal radiographs and CT to definitively exclude vertebral fractures. Still, the lack of extraparenchymal hemorrhage and perivertebral muscle abnormalities makes a concurrent vertebral fracture unlikely in this case.

Other limitations to this case report include the lack of further diagnostic tests for the evaluation of the brachial plexus injury (e.g., electromyography, nerve conduction testing) and lack of histopathological confirmation.

In conclusion, we reported the MRI based diagnosis of traumatic hemorrhagic cervical myelopathy in a dog. Conservative management may be considered in such cases, though the role of surgical treatment (including durotomy) deserves attention and consideration in future studies and cases.

## Data availability statement

The original contributions presented in the study are included in the article/supplementary material, further inquiries can be directed to the corresponding author.

## Ethics statement

Ethical approval was not required for the studies involving animals in accordance with the local legislation and institutional requirements because the animal was treated in accordance with the local legislation and institutional requirements. Written informed consent was obtained from the owners for the participation of their animals in this study.

## Author contributions

KS: Conceptualization, Funding acquisition, Investigation, Visualization, Writing – original draft, Writing – review & editing. IC: Visualization, Writing – review & editing. SP: Conceptualization, Investigation, Visualization, Writing – review & editing.
